# Limited Added Value of Oropharyngeal Swabs for Detecting Pneumococcal Carriage in Adults

**DOI:** 10.1093/ofid/ofaa368

**Published:** 2020-08-18

**Authors:** Jennifer L Farrar, Herine Odiembo, Arthur Odoyo, Godfrey Bigogo, Lindsay Kim, Fernanda C Lessa, Daniel R Feikin, Robert F Breiman, Cynthia G Whitney, Maria G Carvalho, Fabiana C Pimenta

**Affiliations:** 1 Division of Bacterial Diseases, National Center for Immunization and Respiratory Diseases, Centers for Disease Control and Prevention, Atlanta, Georgia, USA; 2 Global Disease Detection Division (GDD) and International Emerging Infections Program (IEIP), Kenya Medical Research Institute (KEMRI)/CDC Public Health and Research Collaboration, Kisumu, Kenya; 3 Emory Global Health Institute, Emory University, Atlanta, Georgia, USA

**Keywords:** adults, carriage, colonization, nasopharyngeal, oropharyngeal, *Streptococcus pneumoniae*

## Abstract

We compared pneumococcal isolation rates and evaluated the benefit of using oropharyngeal (OP) specimens in addition to nasopharyngeal (NP) specimens collected from adults in rural Kenya. Of 846 adults, 52.1% were colonized; pneumococci were detected from both NP and OP specimens in 23.5%, NP only in 22.9%, and OP only in 5.7%. Ten-valent pneumococcal conjugate vaccine strains were detected from both NP and OP in 3.4%, NP only in 4.1%, and OP only in 0.7%. Inclusion of OP swabs increased carriage detection by 5.7%; however, the added cost of collecting and processing OP specimens may justify exclusion from future carriage studies among adults.

*Streptococcus pneumoniae* (pneumococcus) is a highly adaptive bacterium, with the ability to shift from a commensal to pathogenic relationship with the mucosal cells of the human upper respiratory tract (URT), resulting in severe disease when invading other sites. The nasopharynx is considered the major reservoir of pneumococci [1]. Despite reductions in disease burden with the availability of pneumococcal conjugate vaccines (PCV) in 2000, pneumococcus remains the most common cause of bacterial pneumonia in children [2], and the prevalence of antimicrobial-resistant strains remains a concern. Monitoring pneumococcal carriage prevalence and serotype distribution can provide insights into the disease epidemiology, help assess the direct and indirect effects of PCV programs, and predict serotype replacement and antimicrobial resistance evolution. Previously carriage prevalence culture–based studies in adults have described differing colonization rates that could be underestimated due to pneumococci-recovering challenges [1]. However, use of quantitative polymerase chain reaction (qPCR) for detecting pneumococcal serotypes directly from URT samples, especially oropharyngeal (OP) specimens, may overestimate carriage prevalence because of cross-reaction with conserved genes from evolutionarily close species such as *Streptococcus mitis, Streptococcus oralis,* and *Streptococcus infantis* [3, 4]. Therefore, culture-based methods continue to be the gold standard for pneumococcal carriage when OP specimens are used.

Nasopharyngeal (NP) specimens are considered the gold standard [1] for pneumococcal colonization detection in children (culture or molecular techniques). Although current guidelines recommend collection of both NP and OP swabs for pneumococcal carriage detection in adults [1], there is no consensus on the ideal specimen(s) for pneumococcal carriage in adults, and previous studies have yielded conflicting results [1, 5, 6]. OP specimen culture yields much more species diversity than NP, requiring much more proficiency and effort in recovering pneumococcal. Additionally, to our knowledge, no data exist that would suggest that specific serotypes or resistant pneumococcal strains are more or less likely to be found in the oropharynx. We evaluated the potential added benefit of collecting OP specimens in addition to NP specimens for measuring pneumococcal carriage among adults in Kenya.

## METHODS

### Study Design

This analysis was conducted as part of a multiyear cross-sectional study evaluating 10-valent PCV (PCV10) impact on pneumococcal carriage in children and adults in Kenya. Details of the study have been previously described [7, 8]. In summary, pneumococcal carriage surveys were conducted in 2009 and 2011–2013 with a focus on HIV-infected adults. For the analysis presented here, we assessed pneumococcal carriage from NP and OP specimens collected from adults who were parents of children <5 years old enrolled in the 2012 survey, 1 year after PCV10 introduction.

Adults were recruited from Lwak, a rural community with high HIV prevalence rates, part of ongoing health and demographic surveillance and population-based infectious disease surveillance platforms [9]. The platforms were utilized to identify compounds with at least 1 HIV-infected adult and 1 child aged <5 years living in the same household; all adults residing in these households, regardless of HIV status, were invited to participate. After obtaining consent, study staff administered a questionnaire containing data on demographics and risk factors and collected NP and OP specimens from participants.

### Specimen Collection and Laboratory Methods

For each adult, trained staff collected specimens from the nasopharynx (calcium alginate swabs) and from the oropharynx (polyester-tipped swabs). The swabs were placed in separate vials containing STGG (skim milk, tryptone, glucose, glycerin) media, vortexed, and stored at –70°C at the Kenya Medical Research Institute (KEMRI) laboratory in Kisumu, Kenya. They were later transferred to the Streptococcus Laboratory at the Centers for Disease Control and Prevention (CDC) in Atlanta for processing. Pneumococci were isolated from STGG-NP and STGG-OP by inoculating 200 µL into 5 mL of Todd-Hewitt broth with 0.5% yeast extract plus 1 mL of rabbit serum (enrichment) [10]. Pneumococcal isolates were serotyped by Quellung reaction, and serotypes were classified as PCV10 serotypes (1, 4, 5, 6B, 7F, 9V, 14, 18C, 19F, 23F), PCV13 serotypes (PCV10 serotypes plus 3, 6A, and 19A), or nonvaccine type (NVT; all other serotypes). When more than 1 potential pneumococcal colony type was identified, each colony morphology was tested. If multiple serotypes were identified from a specimen, each unique serotype was included in the analysis.

### Data Analysis

We calculated frequencies of basic demographics among the study population. We compared pneumococcal isolation rates using NP and OP specimens and calculated the absolute value added of use of OP for measuring overall and PCV10-type pneumococcal carriage detection. Absolute value added of pneumococcal detection by OPs was calculated as the difference in the overall prevalence of pneumococcal carriage using any specimen type and the prevalence of pneumococcal carriage detected from either both OP and NP swabs or NP swabs only. Statistical significance was assessed using the McNemar test and was defined as a *P* value of <.05. All analyses were done using SAS 9.4 (SAS Institute, Cary, NC, USA).

### Patient Consent Statement

Written consent was obtained from all participants. The design of the work was approved by the KEMRI Ethical Review Committee (SSC-Protocol-1521) and CDC Ethics Committee (Protocol-5594).

## RESULTS

### Study Population

In 2012, NP and OP specimens were collected from 857 adults. Eleven adults were excluded from analysis because specimens collected yielded insufficient volume for culture. Of the remaining 846 adults, the median age (range) was 33.4 (16.4–72.4) years, and 63.4% were female. Of those tested, 79.8% (n = 520/652) were HIV-positive. A total of 61.1% (n = 436/713) and 38.8% (n = 277/713) of adults had 1 child and ≥2 children aged <5 years residing in the same house, respectively. Almost all used wood as the fuel source (97.9%); most depended on subsistence farming; 21.7% (n = 184) of subjects were employed, and 6.1% (n = 52) smoked tobacco.

### Carriage Rates

The overall prevalence of pneumococcal carriage was 52.1% (n = 441), with pneumococci isolated from both NP and OP swabs in 23.5% (n = 199), NP only in 22.9% (n = 194), and OP only in 5.7% (n = 48) (Table 1). Therefore, OP sensitivity for pneumococcal isolation was 56%. Among participants with isolation from both NP and OP swabs, 11% (22/199) of pneumococcal serotypes were discordant. Serotype 3 was the most frequently recovered serotype (13%, 57/441), and serotype 23F was the most frequent PCV10 type (3.4%, 15/441), regardless of swab type. Of 70 adults with PCV10-type pneumococcal isolation from NP or OP specimens, 41.4% (n = 29/70) had PCV10 serotypes detected on both NP and OP swabs, 50% (n = 35/70) on NP only, and 8.6% (n = 6/70) on OP only. Carriage of the additional PCV13 types (3, 6A, and 19A) from NP or OP swabs was 17.7% (n = 78/441).

**Table 1.** Number and Percentage of Pneumococcal Colonization Among Participating Adults, by Specimen Type

Swab Type	No. (%) With Pneumococcal Growth (n = 846)	No. (%) of Swabs Positive for PCV10 Carriage (n = 846)	No. (%) of Swabs Positive for PCV13-Only Carriage (3, 6A, 19A) (n = 846)
NP and OP positive	199 (23.5)	29 (3.4)	41 (4.8)
NP positive only	194 (22.9)	35 (4.1)	29 (3.4)
OP positive only	48 (5.7)	6 (0.7)	8 (0.9)
Negative	405 (47.9)	—	—

Abbreviations: NP, nasopharyngeal; OP, oropharyngeal; PCV, pneumococcal conjugate vaccine.

**Figure 1.**
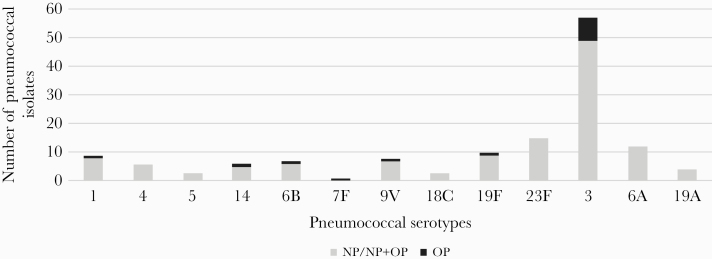
Added value of using oropharyngeal swabs on vaccine-type serotype distribution of pneumococcal isolates in adults in rural Kenya, 2012. Abbreviations: NP, nasopharyngeal; OP, oropharyngeal.

Inclusion of OP swab results increased the overall pneumococcal carriage rate found using NP only by 5.7% (from 46.5% [393/846] to 52.1% [441/846]; *P* < .01) (Table 1). The inclusion of OP swabs increased the PCV10-type carriage rate by 0.7% (from 7.3% [62/846] to 8.3% [70/846]; *P* = .46) (Figure 1).

## DISCUSSION

We found that use of NP swabs was more sensitive than use of OP swabs for detecting pneumococcal carriage in adults. Inclusion of results from OP swabs yielded a small increase in overall detection. This same finding was not observed for PCV10-type carriage, but the overall frequency of PCV10 types was lower and statistical significance was not expected. Of note, we calculated the absolute increase in yield from OP swabs, as calculating the percent change in yield would vary based on the overall pneumococcal carriage rate.

Prior studies have compared pneumococcal carriage detection in adults using NP and OP swabs, with most findings showed greater sensitivity from use of NP swabs. The differences in study findings are possibly due to differences in the methodology applied [5, 6, 11–13]. Satzke et al. examined the results of 5 studies that compared the sensitivity of sampling the nasopharynx and oropharynx of adults using culture only and found that NP swabs were more sensitive in 4 studies [1]. In the Netherlands, they evaluated pneumococcal carriage among parents of 2-year-old children and observed higher yield from NP swabs (18%) than OP (4%) using culture [6]. A randomized controlled trial for 7-valent PCV found pneumococcal carriage to be higher from culture of NP swabs (11.1%) than OP (5.8%) from adults living in the same household with children, and few persons had pneumococcal isolation from both specimens (1.7%); the use of OP swabs increased overall detection by 4.1% (11.1%–15.2%) [13]. Although it could be argued that the high HIV positivity rate could limit the generalizability of the study we present here, our previous cross-sectional study of noninstitutionalized adults aged ≥65 years in the United States found conceptually similar data. In this study, 1.5% (45/2989) carried pneumococci based on NP and OP cultures. Of those, pneumococcus was isolated from 1.2% (n = 35) using NP swabs only, 0.1% (n = 4) from OP swabs only, and 0.2% (n = 6) from both sites, showing almost 10 times greater sensitivity of NP swabs for isolation [14]. Our study observed greater pneumococcal yield from NP swabs over OP swabs using culture, which aligns with findings from previous culture-based studies. The greater yield of pneumococcus from NP specimens could be due to several factors. The oropharynx tends to have greater quantity and variety of bacterial flora, which could mask pneumococcal isolation, leading to misidentification or potentially suppressing growth of pneumococci in the oropharynx.

Use of culture to detect pneumococcal carriage is currently recommended, although the differentiation of *S. pneumoniae* colonies from another closely related group, *mitis* group species, is not simple. Several carriage studies have used molecular methods and found that OP sampling was more sensitive than NP sampling in detecting pneumococci [5, 6]. However, qPCR-based tests for pneumococcal serotyping from both NP and OP swabs have shown that nonpneumococcal streptococci species confounded pneumococcal serotyping using qPCR (by misidentifying streptococci as pneumococci), especially on OP swabs, as they have greater biodiversity [3, 4, 15]. The detection of nonpneumococcal *mitis* group strains using qPCR for serotype/serogroup capsule genes could be a possible explanation for the difference in findings between previous studies showing superiority of OP swabs when compared with NP swabs and our study, which found higher detection for pneumococcal carriage when using NP swabs.

Since pneumococcal carriage studies are important tools for evaluating the impact of PCVs and monitoring the prevalence of drug-resistant vaccine-type pneumococci, optimal methods for specimen collection and detection are needed to reduce variability in the protocols applied to adult pneumococcal colonization studies. The current gold standard for the detection of pneumococcal carriage is the isolation of bacteria from culture [1]; adding the proper broth enrichment step (Todd-Hewitt broth with yeast extract, rabbit serum) to bacterial isolation has been shown to increase recovery rates from respiratory specimens [10]. Currently, *lyt*A qPCR is widely used for pneumococcal molecular detection, despite the fact that a small number of nonpneumococcus isolates may lead to false results; a previous study described only 3 (0.2%) out of 1500 isolates tested using this methodology [16]. Our study yielded a higher detection of pneumococcal carriage from NP swabs than OP swabs using culture and aligns with findings from previous studies that also used culture methods. We suggest that culture for pneumococcal detection should avoid combining NP + OP specimens, as OP specimens are a richer source of nonpneumococcal species that could adversely affect detection of pneumococcal isolates. Potentially more troubling is the use of pneumococcal carriage survey data that rely on qPCR from OP and other oral specimens, which are more likely to overestimate pneumococcal carriage and vaccine type rates including key vaccine types such as serotype 1 [15] and serotype 5 [4].

The large proportion (80%) of HIV-infected adults enrolled in the study by design limits the generalizability of our findings. Nevertheless, collection of OP swabs in addition to NP swabs in adults has little added value, considering the substantial increases in subject discomfort, staff time, laboratory supplies, and cost for sample processing. The OP swabs identified an additional 5.7% increase in pneumococcal carriage in adults vs using NP swabs alone; including OP swabs only increased the yield by 0.7% and 1.6% when compared with NP for detecting PCV10 type and PCV-13 carriage, respectively. Although the inclusion of OP swabs minimally increased carriage detection, the added cost of collecting and processing, the difficulties in isolating pneumococci due to confounding organisms, and the low yield of OP specimens when measuring pneumococcal carriage prevalence might not yield sufficient additional information beyond NP swabs to warrant their inclusion in future carriage studies among adults.

## Notes

***Prior presentation. ***This work has previously been presented at the 11th International Symposium on Pneumococcus and Pneumococcal Disease; April 15–20, 2018; Melbourne, Australia.
